# 4,6-Dimethyl-2-(naphthalen-1-yl)pyrimidine

**DOI:** 10.1107/S1600536811027875

**Published:** 2011-07-23

**Authors:** Xiao-Xue Zhang, Deng-Yong Zhu, Xin-Qi Hao, Xin-Ming Dong, Mao-Ping Song

**Affiliations:** a Department of Chemistry, Henan Key Laboratory of Chemical Biology and Organic Chemistry, Zhengzhou University, Zhengzhou 450052, People’s Republic of China

## Abstract

The asymmetric unit of the title compound, C_16_H_14_N_2_, contains two independent mol­ecules in which the dihedral angles between the pyrimidine and naphthaline rings are 38.20 (5) and 39.35 (5)°. Inter­molecular C—H⋯π contacts and π–π stacking inter­actions [centroid–centroid distances = 3.766 (1) and 3.792 (1) Å] are present in the crystal structure.

## Related literature

For cyclo­metalated Ir(III) complexes, see: Chen *et al.* (2010 ▶); Talarico *et al.* (2010 ▶); Xu *et al.* (2011 ▶); Yang *et al.* (2006 ▶). For the synthesis, see: Wang *et al.* (2011 ▶).
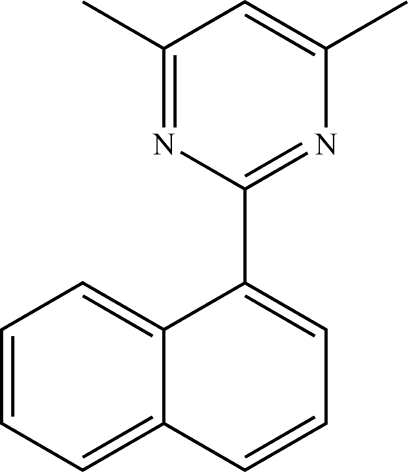


## Experimental

### 

#### Crystal data

C_16_H_14_N_2_
*M* *_r_* = 234.29
Monoclinic, 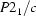

*a* = 14.9022 (18) Å
*b* = 11.4756 (14) Å
*c* = 15.9499 (19) Å
β = 111.028 (1)°
*V* = 2546.0 (5) Å^3^
*Z* = 8
Mo *K*α radiation
μ = 0.07 mm^−1^
*T* = 296 K
0.50 × 0.37 × 0.29 mm

#### Data collection

Bruker SMART APEX CCD area-detector diffractometer
Absorption correction: multi-scan (*SADABS*; Sheldrick, 1996 ▶) *T*_min_ = 0.965, *T*_max_ = 0.979
18638 measured reflections
4733 independent reflections
3442 reflections with *I* > 2σ(*I*)
*R*_int_ = 0.023

#### Refinement

*R*[*F* ^2^ > 2σ(*F* ^2^)] = 0.044
*wR*(*F* ^2^) = 0.129
*S* = 1.06
4733 reflections
329 parameters
H-atom parameters constrained
Δρ_max_ = 0.24 e Å^−3^
Δρ_min_ = −0.18 e Å^−3^

### 

Data collection: *APEX2* (Bruker, 2004 ▶); cell refinement: *SAINT* (Bruker, 2004 ▶); data reduction: *SAINT*; program(s) used to solve structure: *SHELXS97* (Sheldrick, 2008 ▶); program(s) used to refine structure: *SHELXL97* (Sheldrick, 2008 ▶); molecular graphics: *SHELXTL* (Sheldrick, 2008 ▶); software used to prepare material for publication: *SHELXL97* and *PLATON* (Spek, 2009 ▶).

## Supplementary Material

Crystal structure: contains datablock(s) global, I. DOI: 10.1107/S1600536811027875/si2364sup1.cif

Structure factors: contains datablock(s) I. DOI: 10.1107/S1600536811027875/si2364Isup2.hkl

Supplementary material file. DOI: 10.1107/S1600536811027875/si2364Isup3.cml

Additional supplementary materials:  crystallographic information; 3D view; checkCIF report

## Figures and Tables

**Table 1:** Hydrogen-bond geometry (Å, °) *Cg*1, *Cg*2, *Cg*5 and *Cg*6 are the centroids of the N1/N2/C11–C14, C1/C2/C7–C10, N3/N4,C27–C30 and C17–C21/C26 rings, respectively.

*D*—H⋯*A*	*D*—H	H⋯*A*	*D*⋯*A*	*D*—H⋯*A*
C22—H22⋯*Cg*1^i^	0.93	2.74	3.651 (4)	167
C13—H13⋯*Cg*2^ii^	0.93	2.76	3.597 (4)	150
C6—H6⋯*Cg*5	0.93	2.75	3.665 (2)	168
C29—H29⋯*Cg*6^iii^	0.93	2.72	3.546 (3)	149

Symmetry codes: (i) 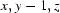; (ii) 
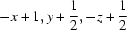
; (iii) 
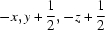
.

**Fig. 1.**
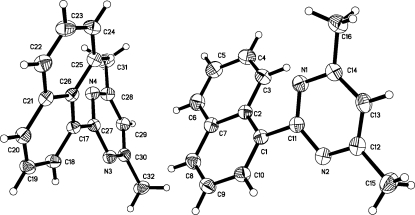
The molecular structure of the title compound with displacement ellipsoids at the 30% probability level.

## supplementary crystallographic information

### Comment

In recent years, various types of cyclometalated Ir(III) complexes have been developed, such as homoleptic complexes, heteroleptic neutral complexes and cationic complexes (Chen *et al.*, 2010; Talarico *et al.*, 2010). In contrast to the most phenylpyridine ligands, few examples of naphthalenylpyrimidine iridium complexes have been reported (Xu *et al.*, 2011; Yang *et al.*, 2006). The title compound was obtained from the Suzuki coupling reaction with 1-naphthaleneboronic acid and 2-iodo-4,6-dimethylpyrimidine.

The crystal structure analysis of the title compound revealed that the asymmetric unit consists of two independent molecules (Fig. 1). The pyrimidine ring and naphthaline ring are not coplanar with the dihedral angles of 38.20 (5)° and 39.35 (5)°. All the bond distances and angles are within normal ranges. In the crystal of title compound there exist intermolecular C—H···π interactions (Table 1) and π—π stacking interactions [centroid-centroid distances *Cg*1 (N1,C11,N2,C12,C13,C14)···*Cg*7^ii^ (C21–C26) and *Cg*3 (C2–C7)···*Cg*5^iii^ (N3,C27,N4,C28,C29,C30) are 3.7659 (12) and 3.7915 (12) Å, the perpendicular distances *Cg*1 on *Cg*7^ii^ and *Cg*3 on *Cg*5^iii^ are 3.4761 (7) and 3.5318 (8) Å, respectively; [symmetry codes: ii = 1 - *x*, 1/2 + *y*, 1/2 - *z*; iii = - *x*, 1/2 + *y*, 1/2 - *z*] (see also Table 1). Cg2 and Cg6 are the centroids of the six-membered rings of the independent naphthyl units (C1,C2,C7,C8,C9,C10) and (C17 - C21,C26), respectively.

### Experimental

The title compound was obtained from the coupling reaction of 1-naphthaleneboronic acid and 2-iodo-4,6-dimethylpyrimidine as described in literature (Wang *et al.*, 2011) and recrystallized from dichloromethane- petroleum ether solution at room temperature to give the desired crystals suitable for single-crystal X-ray diffraction.

### Refinement

H atoms attached to C atoms of the title compound were placed in geometrically idealized positions and treated as riding with C—H distances constrained to 0.93–0.96 Å, and with *U*_iso_(H)=1.2^.^*U*_eq_(C), 1.5^.^*U*_eq_(methyl H).

### Crystal data

**Table:**

C_16_H_14_N_2_	*F*(000) = 992
*M**_r_* = 234.29	*D*_x_ = 1.222 Mg m^−^^3^
Monoclinic, *P*2_1_/*c*	Mo *K*α radiation, λ = 0.71073 Å
*a* = 14.9022 (18) Å	Cell parameters from 5588 reflections
*b* = 11.4756 (14) Å	θ = 2.4–27.4°
*c* = 15.9499 (19) Å	µ = 0.07 mm^−^^1^
β = 111.028 (1)°	*T* = 296 K
*V* = 2546.0 (5) Å^3^	Block, colourless
*Z* = 8	0.50 × 0.37 × 0.29 mm

### Data collection

**Table:**

Bruker SMART APEX CCD area-detector diffractometer	4733 independent reflections
Radiation source: fine-focus sealed tube	3442 reflections with *I* > 2σ(*I*)
graphite	*R*_int_ = 0.023
φ and ω scans	θ_max_ = 25.5°, θ_min_ = 2.4°
Absorption correction: multi-scan (*SADABS*; Sheldrick, 1996)	*h* = −18→18
*T*_min_ = 0.965, *T*_max_ = 0.979	*k* = −13→13
18638 measured reflections	*l* = −19→19

### Refinement

**Table:**

Refinement on *F*^2^	Primary atom site location: structure-invariant direct methods
Least-squares matrix: full	Secondary atom site location: difference Fourier map
*R*[*F*^2^ > 2σ(*F*^2^)] = 0.044	Hydrogen site location: inferred from neighbouring sites
*wR*(*F*^2^) = 0.129	H-atom parameters constrained
*S* = 1.06	*w* = 1/[σ^2^(*F*_o_^2^) + (0.0523*P*)^2^ + 0.8594*P*] where *P* = (*F*_o_^2^ + 2*F*_c_^2^)/3
4733 reflections	(Δ/σ)_max_ < 0.001
329 parameters	Δρ_max_ = 0.24 e Å^−^^3^
0 restraints	Δρ_min_ = −0.18 e Å^−^^3^

### Special details

**Table:**

Geometry. All e.s.d.'s (except the e.s.d. in the dihedral angle between two l.s. planes)are estimated using the full covariance matrix. The cell e.s.d.'s are takeninto account individually in the estimation of e.s.d.'s in distances, anglesand torsion angles; correlations between e.s.d.'s in cell parameters are onlyused when they are defined by crystal symmetry. An approximate (isotropic)treatment of cell e.s.d.'s is used for estimating e.s.d.'s involving l.s. planes.
Refinement. Refinement of *F*^2^ against ALL reflections. The weighted *R*-factor *wR* and goodness of fit *S* are based on *F*^2^, conventional *R*-factors *R* are based on *F*, with *F* set to zero for negative *F*^2^. The threshold expression of *F*^2^ > σ(*F*^2^) is used only for calculating *R*-factors(gt) *etc*. and is not relevant to the choice of reflections for refinement. *R*-factors based on *F*^2^ are statistically about twice as large as those based on *F*, and *R*- factors based on ALL data will be even larger.

### Fractional atomic coordinates and isotropic or equivalent isotropic displacement parameters (Å^2^)

**Table:**

	*x*	*y*	*z*	*U*_iso_*/*U*_eq_	
C1	0.33022 (12)	0.56804 (15)	0.19201 (11)	0.0391 (4)	
C2	0.28518 (12)	0.54003 (15)	0.25537 (11)	0.0382 (4)	
C3	0.31319 (13)	0.58798 (17)	0.34283 (12)	0.0466 (5)	
H3	0.3632	0.6417	0.3614	0.056*	
C4	0.26816 (14)	0.55681 (19)	0.40020 (13)	0.0545 (5)	
H4	0.2882	0.5891	0.4575	0.065*	
C5	0.19230 (15)	0.47692 (19)	0.37416 (14)	0.0574 (5)	
H5	0.1631	0.4549	0.4144	0.069*	
C6	0.16151 (14)	0.43166 (18)	0.29020 (14)	0.0535 (5)	
H6	0.1102	0.3797	0.2729	0.064*	
C7	0.20588 (12)	0.46173 (16)	0.22806 (12)	0.0429 (4)	
C8	0.17279 (13)	0.41556 (17)	0.14022 (13)	0.0493 (5)	
H8	0.1219	0.3630	0.1230	0.059*	
C9	0.21425 (13)	0.44683 (17)	0.08061 (13)	0.0507 (5)	
H9	0.1905	0.4175	0.0224	0.061*	
C10	0.29303 (12)	0.52348 (16)	0.10659 (12)	0.0453 (4)	
H10	0.3206	0.5445	0.0649	0.054*	
C11	0.41918 (12)	0.63937 (15)	0.21483 (12)	0.0399 (4)	
C12	0.50852 (13)	0.76663 (16)	0.16641 (13)	0.0466 (4)	
C13	0.58104 (13)	0.75808 (17)	0.24906 (13)	0.0481 (5)	
H13	0.6379	0.7995	0.2612	0.058*	
C14	0.56827 (13)	0.68750 (16)	0.31349 (12)	0.0450 (4)	
C15	0.51631 (16)	0.8421 (2)	0.09295 (15)	0.0643 (6)	
H15A	0.4803	0.8079	0.0358	0.097*	
H15B	0.5826	0.8489	0.0991	0.097*	
H15C	0.4910	0.9180	0.0968	0.097*	
C16	0.64365 (14)	0.67278 (19)	0.40496 (13)	0.0588 (5)	
H16A	0.6343	0.7300	0.4450	0.088*	
H16B	0.7061	0.6828	0.4014	0.088*	
H16C	0.6388	0.5962	0.4271	0.088*	
C17	0.12658 (11)	0.06318 (15)	0.19754 (11)	0.0357 (4)	
C18	0.09762 (13)	0.01305 (17)	0.11404 (11)	0.0435 (4)	
H18	0.0368	0.0304	0.0729	0.052*	
C19	0.15642 (13)	−0.06341 (16)	0.08848 (12)	0.0454 (4)	
H19	0.1345	−0.0962	0.0313	0.054*	
C20	0.24584 (13)	−0.08964 (16)	0.14757 (12)	0.0440 (4)	
H20	0.2842	−0.1420	0.1311	0.053*	
C21	0.28081 (12)	−0.03786 (15)	0.23378 (11)	0.0381 (4)	
C22	0.37491 (13)	−0.06205 (18)	0.29471 (13)	0.0501 (5)	
H22	0.4136	−0.1140	0.2783	0.060*	
C23	0.40930 (13)	−0.0107 (2)	0.37641 (13)	0.0561 (5)	
H23	0.4709	−0.0283	0.4159	0.067*	
C24	0.35244 (13)	0.06895 (18)	0.40169 (12)	0.0500 (5)	
H24	0.3771	0.1048	0.4576	0.060*	
C25	0.26154 (12)	0.09449 (16)	0.34545 (11)	0.0413 (4)	
H25	0.2250	0.1479	0.3634	0.050*	
C26	0.22168 (11)	0.04068 (14)	0.25978 (10)	0.0345 (4)	
C27	0.05443 (11)	0.13401 (15)	0.21966 (11)	0.0360 (4)	
C28	−0.01736 (12)	0.18418 (16)	0.31890 (12)	0.0425 (4)	
C29	−0.08211 (12)	0.25082 (16)	0.25319 (12)	0.0432 (4)	
H29	−0.1305	0.2911	0.2649	0.052*	
C30	−0.07448 (12)	0.25720 (15)	0.16983 (12)	0.0407 (4)	
C31	−0.02058 (15)	0.1730 (2)	0.41097 (13)	0.0608 (6)	
H31A	0.0023	0.0973	0.4347	0.091*	
H31B	−0.0855	0.1828	0.4082	0.091*	
H31C	0.0195	0.2317	0.4493	0.091*	
C32	−0.14053 (14)	0.33086 (18)	0.09602 (13)	0.0548 (5)	
H32A	−0.1143	0.4079	0.0998	0.082*	
H32B	−0.2023	0.3348	0.1019	0.082*	
H32C	−0.1473	0.2971	0.0390	0.082*	
N1	0.48627 (10)	0.62722 (13)	0.29660 (10)	0.0437 (4)	
N2	0.42607 (10)	0.70672 (13)	0.14848 (10)	0.0443 (4)	
N3	−0.00560 (10)	0.19787 (12)	0.15203 (9)	0.0395 (3)	
N4	0.05243 (10)	0.12487 (13)	0.30227 (9)	0.0406 (4)	

### Atomic displacement parameters (Å^2^)

**Table:**

	*U*^11^	*U*^22^	*U*^33^	*U*^12^	*U*^13^	*U*^23^
C1	0.0340 (9)	0.0402 (10)	0.0421 (10)	0.0027 (7)	0.0125 (7)	0.0004 (8)
C2	0.0324 (8)	0.0386 (10)	0.0423 (10)	0.0043 (7)	0.0119 (7)	0.0018 (7)
C3	0.0382 (9)	0.0543 (12)	0.0478 (11)	−0.0007 (8)	0.0160 (8)	−0.0062 (9)
C4	0.0525 (12)	0.0654 (13)	0.0480 (11)	0.0035 (10)	0.0212 (9)	−0.0036 (10)
C5	0.0562 (12)	0.0655 (14)	0.0593 (13)	−0.0010 (10)	0.0315 (10)	0.0055 (11)
C6	0.0465 (11)	0.0546 (12)	0.0631 (13)	−0.0071 (9)	0.0240 (10)	0.0014 (10)
C7	0.0358 (9)	0.0429 (10)	0.0493 (11)	0.0013 (8)	0.0145 (8)	0.0025 (8)
C8	0.0402 (10)	0.0505 (12)	0.0530 (11)	−0.0065 (8)	0.0118 (9)	−0.0033 (9)
C9	0.0485 (11)	0.0533 (12)	0.0447 (11)	−0.0035 (9)	0.0100 (9)	−0.0068 (9)
C10	0.0433 (10)	0.0496 (11)	0.0437 (10)	0.0003 (8)	0.0165 (8)	0.0014 (8)
C11	0.0382 (9)	0.0386 (10)	0.0453 (10)	0.0041 (7)	0.0178 (8)	−0.0016 (8)
C12	0.0456 (10)	0.0429 (11)	0.0573 (12)	0.0013 (8)	0.0257 (9)	−0.0002 (9)
C13	0.0406 (10)	0.0461 (11)	0.0606 (12)	−0.0068 (8)	0.0220 (9)	−0.0064 (9)
C14	0.0396 (9)	0.0449 (11)	0.0516 (11)	0.0005 (8)	0.0179 (8)	−0.0064 (9)
C15	0.0676 (14)	0.0621 (14)	0.0678 (14)	−0.0064 (11)	0.0298 (11)	0.0121 (11)
C16	0.0483 (11)	0.0662 (14)	0.0560 (12)	−0.0050 (10)	0.0115 (9)	−0.0046 (10)
C17	0.0349 (9)	0.0390 (9)	0.0348 (9)	0.0030 (7)	0.0143 (7)	0.0021 (7)
C18	0.0406 (9)	0.0518 (11)	0.0356 (9)	0.0068 (8)	0.0108 (8)	−0.0008 (8)
C19	0.0510 (11)	0.0490 (11)	0.0377 (10)	0.0040 (9)	0.0179 (8)	−0.0056 (8)
C20	0.0470 (10)	0.0450 (11)	0.0468 (10)	0.0067 (8)	0.0251 (8)	−0.0020 (8)
C21	0.0360 (9)	0.0390 (10)	0.0433 (10)	0.0033 (7)	0.0190 (8)	0.0053 (7)
C22	0.0404 (10)	0.0549 (12)	0.0557 (12)	0.0135 (9)	0.0182 (9)	0.0046 (9)
C23	0.0373 (10)	0.0699 (14)	0.0528 (12)	0.0095 (10)	0.0061 (9)	0.0055 (10)
C24	0.0425 (10)	0.0611 (13)	0.0412 (10)	−0.0032 (9)	0.0086 (8)	−0.0025 (9)
C25	0.0380 (9)	0.0453 (10)	0.0418 (10)	0.0000 (8)	0.0159 (8)	−0.0017 (8)
C26	0.0340 (8)	0.0365 (9)	0.0366 (9)	0.0012 (7)	0.0169 (7)	0.0035 (7)
C27	0.0333 (8)	0.0366 (9)	0.0375 (9)	0.0001 (7)	0.0121 (7)	−0.0011 (7)
C28	0.0379 (9)	0.0501 (11)	0.0412 (10)	0.0036 (8)	0.0163 (8)	−0.0003 (8)
C29	0.0380 (9)	0.0469 (11)	0.0458 (10)	0.0099 (8)	0.0165 (8)	−0.0017 (8)
C30	0.0381 (9)	0.0391 (10)	0.0435 (10)	0.0044 (8)	0.0129 (8)	−0.0002 (8)
C31	0.0629 (13)	0.0800 (16)	0.0458 (11)	0.0202 (11)	0.0271 (10)	0.0076 (10)
C32	0.0602 (12)	0.0546 (12)	0.0471 (11)	0.0209 (10)	0.0162 (9)	0.0075 (9)
N1	0.0383 (8)	0.0447 (9)	0.0482 (9)	−0.0011 (7)	0.0158 (7)	−0.0022 (7)
N2	0.0426 (8)	0.0431 (9)	0.0499 (9)	0.0018 (7)	0.0200 (7)	0.0003 (7)
N3	0.0381 (8)	0.0402 (8)	0.0399 (8)	0.0045 (6)	0.0136 (6)	0.0015 (6)
N4	0.0377 (8)	0.0465 (9)	0.0392 (8)	0.0066 (7)	0.0159 (6)	0.0019 (7)

### Geometric parameters (Å, °)

**Table:**

C1—C10	1.372 (2)	C17—C18	1.371 (2)
C1—C2	1.436 (2)	C17—C26	1.432 (2)
C1—C11	1.488 (2)	C17—C27	1.488 (2)
C2—C3	1.416 (2)	C18—C19	1.400 (2)
C2—C7	1.423 (2)	C18—H18	0.9300
C3—C4	1.362 (3)	C19—C20	1.361 (2)
C3—H3	0.9300	C19—H19	0.9300
C4—C5	1.398 (3)	C20—C21	1.415 (2)
C4—H4	0.9300	C20—H20	0.9300
C5—C6	1.354 (3)	C21—C22	1.418 (2)
C5—H5	0.9300	C21—C26	1.422 (2)
C6—C7	1.417 (3)	C22—C23	1.353 (3)
C6—H6	0.9300	C22—H22	0.9300
C7—C8	1.411 (3)	C23—C24	1.400 (3)
C8—C9	1.355 (3)	C23—H23	0.9300
C8—H8	0.9300	C24—C25	1.361 (2)
C9—C10	1.405 (3)	C24—H24	0.9300
C9—H9	0.9300	C25—C26	1.421 (2)
C10—H10	0.9300	C25—H25	0.9300
C11—N1	1.336 (2)	C27—N4	1.333 (2)
C11—N2	1.344 (2)	C27—N3	1.345 (2)
C12—N2	1.346 (2)	C28—N4	1.347 (2)
C12—C13	1.376 (3)	C28—C29	1.374 (2)
C12—C15	1.495 (3)	C28—C31	1.492 (2)
C13—C14	1.374 (3)	C29—C30	1.376 (2)
C13—H13	0.9300	C29—H29	0.9300
C14—N1	1.345 (2)	C30—N3	1.344 (2)
C14—C16	1.498 (3)	C30—C32	1.496 (2)
C15—H15A	0.9600	C31—H31A	0.9600
C15—H15B	0.9600	C31—H31B	0.9600
C15—H15C	0.9600	C31—H31C	0.9600
C16—H16A	0.9600	C32—H32A	0.9600
C16—H16B	0.9600	C32—H32B	0.9600
C16—H16C	0.9600	C32—H32C	0.9600
			
C10—C1—C2	119.41 (16)	C26—C17—C27	123.63 (14)
C10—C1—C11	117.06 (15)	C17—C18—C19	122.29 (16)
C2—C1—C11	123.47 (15)	C17—C18—H18	118.9
C3—C2—C7	117.77 (16)	C19—C18—H18	118.9
C3—C2—C1	124.01 (16)	C20—C19—C18	119.77 (16)
C7—C2—C1	118.20 (16)	C20—C19—H19	120.1
C4—C3—C2	121.16 (18)	C18—C19—H19	120.1
C4—C3—H3	119.4	C19—C20—C21	120.51 (16)
C2—C3—H3	119.4	C19—C20—H20	119.7
C3—C4—C5	120.89 (19)	C21—C20—H20	119.7
C3—C4—H4	119.6	C20—C21—C22	120.85 (16)
C5—C4—H4	119.6	C20—C21—C26	119.93 (15)
C6—C5—C4	119.76 (18)	C22—C21—C26	119.22 (16)
C6—C5—H5	120.1	C23—C22—C21	120.97 (17)
C4—C5—H5	120.1	C23—C22—H22	119.5
C5—C6—C7	121.38 (19)	C21—C22—H22	119.5
C5—C6—H6	119.3	C22—C23—C24	120.18 (17)
C7—C6—H6	119.3	C22—C23—H23	119.9
C8—C7—C6	121.22 (17)	C24—C23—H23	119.9
C8—C7—C2	119.81 (16)	C25—C24—C23	120.84 (18)
C6—C7—C2	118.97 (17)	C25—C24—H24	119.6
C9—C8—C7	120.82 (17)	C23—C24—H24	119.6
C9—C8—H8	119.6	C24—C25—C26	120.92 (17)
C7—C8—H8	119.6	C24—C25—H25	119.5
C8—C9—C10	120.13 (18)	C26—C25—H25	119.5
C8—C9—H9	119.9	C25—C26—C21	117.83 (15)
C10—C9—H9	119.9	C25—C26—C17	123.81 (15)
C1—C10—C9	121.54 (17)	C21—C26—C17	118.34 (15)
C1—C10—H10	119.2	N4—C27—N3	126.26 (15)
C9—C10—H10	119.2	N4—C27—C17	117.76 (14)
N1—C11—N2	125.94 (16)	N3—C27—C17	115.91 (14)
N1—C11—C1	118.07 (15)	N4—C28—C29	120.77 (16)
N2—C11—C1	115.91 (15)	N4—C28—C31	116.77 (16)
N2—C12—C13	120.69 (17)	C29—C28—C31	122.47 (16)
N2—C12—C15	116.82 (17)	C28—C29—C30	119.18 (16)
C13—C12—C15	122.50 (18)	C28—C29—H29	120.4
C14—C13—C12	119.12 (17)	C30—C29—H29	120.4
C14—C13—H13	120.4	N3—C30—C29	120.73 (16)
C12—C13—H13	120.4	N3—C30—C32	117.18 (16)
N1—C14—C13	120.87 (17)	C29—C30—C32	122.09 (16)
N1—C14—C16	116.57 (17)	C28—C31—H31A	109.5
C13—C14—C16	122.56 (17)	C28—C31—H31B	109.5
C12—C15—H15A	109.5	H31A—C31—H31B	109.5
C12—C15—H15B	109.5	C28—C31—H31C	109.5
H15A—C15—H15B	109.5	H31A—C31—H31C	109.5
C12—C15—H15C	109.5	H31B—C31—H31C	109.5
H15A—C15—H15C	109.5	C30—C32—H32A	109.5
H15B—C15—H15C	109.5	C30—C32—H32B	109.5
C14—C16—H16A	109.5	H32A—C32—H32B	109.5
C14—C16—H16B	109.5	C30—C32—H32C	109.5
H16A—C16—H16B	109.5	H32A—C32—H32C	109.5
C14—C16—H16C	109.5	H32B—C32—H32C	109.5
H16A—C16—H16C	109.5	C11—N1—C14	116.74 (15)
H16B—C16—H16C	109.5	C11—N2—C12	116.63 (15)
C18—C17—C26	119.09 (15)	C30—N3—C27	116.44 (14)
C18—C17—C27	117.21 (14)	C27—N4—C28	116.61 (14)
			
C10—C1—C2—C3	−175.15 (17)	C21—C22—C23—C24	−0.8 (3)
C11—C1—C2—C3	7.8 (3)	C22—C23—C24—C25	1.1 (3)
C10—C1—C2—C7	3.1 (2)	C23—C24—C25—C26	0.3 (3)
C11—C1—C2—C7	−173.91 (16)	C24—C25—C26—C21	−1.9 (3)
C7—C2—C3—C4	2.5 (3)	C24—C25—C26—C17	179.75 (17)
C1—C2—C3—C4	−179.26 (18)	C20—C21—C26—C25	−177.21 (15)
C2—C3—C4—C5	−0.4 (3)	C22—C21—C26—C25	2.0 (2)
C3—C4—C5—C6	−1.5 (3)	C20—C21—C26—C17	1.3 (2)
C4—C5—C6—C7	1.3 (3)	C22—C21—C26—C17	−179.48 (15)
C5—C6—C7—C8	−179.16 (19)	C18—C17—C26—C25	175.28 (16)
C5—C6—C7—C2	0.8 (3)	C27—C17—C26—C25	−7.9 (3)
C3—C2—C7—C8	177.34 (16)	C18—C17—C26—C21	−3.1 (2)
C1—C2—C7—C8	−1.0 (3)	C27—C17—C26—C21	173.76 (15)
C3—C2—C7—C6	−2.6 (3)	C18—C17—C27—N4	140.10 (17)
C1—C2—C7—C6	179.01 (16)	C26—C17—C27—N4	−36.8 (2)
C6—C7—C8—C9	178.50 (19)	C18—C17—C27—N3	−37.2 (2)
C2—C7—C8—C9	−1.5 (3)	C26—C17—C27—N3	145.92 (16)
C7—C8—C9—C10	1.9 (3)	N4—C28—C29—C30	−0.5 (3)
C2—C1—C10—C9	−2.8 (3)	C31—C28—C29—C30	179.84 (19)
C11—C1—C10—C9	174.40 (16)	C28—C29—C30—N3	1.0 (3)
C8—C9—C10—C1	0.3 (3)	C28—C29—C30—C32	−178.10 (17)
C10—C1—C11—N1	−140.99 (17)	N2—C11—N1—C14	−0.5 (3)
C2—C1—C11—N1	36.1 (2)	C1—C11—N1—C14	176.14 (15)
C10—C1—C11—N2	35.9 (2)	C13—C14—N1—C11	0.2 (2)
C2—C1—C11—N2	−146.97 (16)	C16—C14—N1—C11	−179.46 (16)
N2—C12—C13—C14	−0.1 (3)	N1—C11—N2—C12	0.4 (3)
C15—C12—C13—C14	179.50 (18)	C1—C11—N2—C12	−176.27 (15)
C12—C13—C14—N1	0.0 (3)	C13—C12—N2—C11	−0.1 (2)
C12—C13—C14—C16	179.70 (18)	C15—C12—N2—C11	−179.72 (17)
C26—C17—C18—C19	2.7 (3)	C29—C30—N3—C27	−0.5 (2)
C27—C17—C18—C19	−174.41 (17)	C32—C30—N3—C27	178.65 (16)
C17—C18—C19—C20	−0.2 (3)	N4—C27—N3—C30	−0.6 (3)
C18—C19—C20—C21	−1.7 (3)	C17—C27—N3—C30	176.45 (15)
C19—C20—C21—C22	−178.11 (18)	N3—C27—N4—C28	1.0 (3)
C19—C20—C21—C26	1.1 (3)	C17—C27—N4—C28	−175.97 (15)
C20—C21—C22—C23	178.51 (18)	C29—C28—N4—C27	−0.4 (3)
C26—C21—C22—C23	−0.7 (3)	C31—C28—N4—C27	179.27 (17)

### Hydrogen-bond geometry (Å, °)

**Table:**

Cg1, Cg2, Cg5 and Cg6 are the centroids of the N1/N2/C11–C14, C1/C2/C7–C10, N3/N4,C27–C30 and C17–C21/C26 rings, respectively.

**Table:**

*D*—H···*A*	*D*—H	H···*A*	*D*···*A*	*D*—H···*A*
C22—H22···Cg1^i^	0.93	2.74	3.651 (4)	167
C13—H13···Cg2^ii^	0.93	2.76	3.597 (4)	150
C6—H6···Cg5	0.93	2.75	3.665 (2)	168
C29—H29···Cg6^iii^	0.93	2.72	3.546 (3)	149

Symmetry codes: (i) *x*, *y*−1, *z*; (ii) −*x*+1, *y*+1/2, −*z*+1/2;  (iii) −*x*, *y*+1/2, −*z*+1/2.
